# Genetic architecture of variation in Arabidopsis thaliana rosettes

**DOI:** 10.1371/journal.pone.0263985

**Published:** 2022-02-16

**Authors:** Odín Morón-García, Gina A. Garzón-Martínez, M. J. Pilar Martínez-Martín, Jason Brook, Fiona M. K. Corke, John H. Doonan, Anyela V. Camargo Rodríguez

**Affiliations:** The National Plant Phenomics Centre, Institute of Biological, Rural and Environmental Sciences (IBERS), Aberystwyth University, Aberystwyth, United Kingdom; Jeju National University, REPUBLIC OF KOREA

## Abstract

Rosette morphology across Arabidopsis accessions exhibits considerable variation. Here we report a high-throughput phenotyping approach based on automatic image analysis to quantify rosette shape and dissect the underlying genetic architecture. Shape measurements of the rosettes in a core set of Recombinant Inbred Lines from an advanced mapping population (Multiparent Advanced Generation Inter-Cross or MAGIC) derived from inter-crossing 19 natural accessions. Image acquisition and analysis was scaled to extract geometric descriptors from time stamped images of growing rosettes. Shape analyses revealed heritable morphological variation at early juvenile stages and QTL mapping resulted in over 116 chromosomal regions associated with trait variation within the population. Many QTL linked to variation in shape were located near genes related to hormonal signalling and signal transduction pathways while others are involved in shade avoidance and transition to flowering. Our results suggest rosette shape arises from modular integration of sub-organ morphologies and can be considered a functional trait subjected to selective pressures of subsequent morphological traits. On an applied aspect, QTLs found will be candidates for further research on plant architecture.

## Introduction

Understanding the role of environmental conditions on plant development is of increasing importance to counterbalance climate change effects in agricultural species [1]. Natural variation found in nature can reveal features that are subject to selective pressure, including that exerted by the local environment [2–4]. For example, shoot architecture integrates the interaction between genetic determinants, developmental history, the environment and adaptation to particular lifestyles [5, 6]. The rosette forms during the vegetative phase of many species including several major crops in the Brassicaceae family. The rosette is an attractive model to understand the genetic architecture of variation in plant form thanks to the quantity and quality of genetic and natural resources available in the model species, Arabidopsis [2, 7, 8].

Rosettes display limited internode extension and generally are comprised of a spiral of leaves, overlapping to a greater or lesser extent [9]. This leaf assemblage has recurrently appeared along angiosperm phylogeny [10], yet its adaptive significance remains unclear. Rosettes probably occupy the space for photosynthesis, excluding nearby plants by establishing ground cover [11] while remaining cryptic to larger herbivores [12]. Leaf distribution and size may respond to environmental factors such as light interception [13], herbivore grazing [14], abiotic stress [4, 15]. We rationalise that, over evolutionary time, small changes in leaf size and shape, internode extension, and other developmental processes could have been selected and therefore, shape variation could give insight into the genetic control of the rosette habit.

Shape is a ubiquitous concept in biological research with context dependant morphometrical methodologies [16, 17]. Outline shape descriptors [18–21] define specific and quantitative [22] aspects of form, e.g. roundness [23]. They can be combined with computer vision [24, 25] for high-throughput phenotyping [26] of plants organs, assemblages, full plants and field plots. These are global descriptors [27, 28] with some degree of overlapping [29–31] and dependency [32]. For example, a cogwheel, a starfish-like shape or Arabidopsis rosettes are visually different but with similar roundness scores and different circularity ones, due to their different definition [33, 34].

High dimensional vectors of descriptors need multivariate statistics to discriminate shapes numerically. Particularly, Principal Component Analysis (PCA) [35, 36] build latent variables [37, 38] (or latent shapes [39]) generating a “morphospace” [40–42], with specific mathematical properties [43, 44] e.g. relative warps and synthetic shape axis [45–47]. PCA-based morphospace cleaves univariate morphological features [47] that can be analysed as individual morphological phenotypes [43] in quantitative trait loci (QTL) mapping experiments [48–50]. Also, PCA is used to separate size from shape [51, 52], study allometric growth [41, 53] or when sample size is much smaller than the number of variables measured.

To interrogate shape and size variation over populations large enough to reveal associations with the underlying genetics requires measurement techniques that are scalable. Imaging techniques are readily scalable and have been widely used to measure growth rate [3, 54, 55], leaf number and size [56], leaf hyponasty [57] and hypocotyl angle [58], and can be used to estimate morphological parameters. We have previously characterized rosette morphology in 19 Arabidopsis ecotypes using image based approaches during growth and development [22] and other studies have used similar descriptors for screening large Arabidopsis populations and mutants, tracking morphological changes over time and allowing a more precise dissection of developmental timing of plant growth and development [59–63]. Image analysis can quantify size and shape variation due to defined genetic lesions in rosette plants [64] and used to identify QTL for variation in rosette area, revealing a number of candidate genes for growth and size traits [3, 4, 62, 63, 65–67]. More recently, a multi-scale approach was used with the purpose of linking genes to plant shape at several scales, from the whole plant to cells and tissues attending to quantitative measurements extracted from digital images and models [50]. It can be argued that research on plant morphology and gene mapping would benefit advanced automatic phenotyping methods scalable to large populations [68, 69]. For that reason, it is important to find and describe measurements that ensure an accurate capture of morphological variability with biological meaning.

QTL mapping experiments leverages the phenotypic variation associated with the standing genetic variation. Biparental crosses estimate linkage between markers [70, 71] and between markers and phenotypes e.g. Composite Interval Mapping and Haley-Knot regression [72, 73]. Mapping resolution depends on recombination rate, marker distribution [74, 75] and trait complexity [76]. In contrast, natural populations can harbour higher genetic and phenotypic variation [77], have shorter haplotypic regions due to higher crossover number [78] and may present some population structure that confound analysis. Genome-Wide Association Mapping (GWAS) exploits linkage disequilibrium [79] to associate statistically traits to genotyped markers [80] by ANOVA-like tests [81, 82]. Linear mixed models correct for experimental factors [83–85] like population structure, replication levels, treatments, etc. Multiple testing, for many markers, need False Discovery Rate correcting procedures like Bonferroni or Benjamini-Hochberg.

Advanced mapping populations, like Multiparent Advanced Generation Inter-Cross (MAGIC) [86], trade the advantages and disadvantages of classical mapping populations in terms of resolution and efficiency [87]. These are crosses of up to 20 genetically and phenotypically diverse parental types [88] and several generations of selfing. The strategy reduces population structure and generates small haplotypic mosaics, providing finer resolution mapping than bi-parental with less false positives than natural populations. MAGIC allows haplotype reconstruction for markers alleles as founder-of-origin [89–91] tracking genetic variation back to parental sequence level [92]. Tailored bayesian models [93, 94] improves parameter estimation and permutation-based procedures reduce False Discovery Rates [95].

The use of highly diverse natural accessions increases the opportunity to find trait-associated markers that may not be available in laboratory experimental populations. In particular, the wild-type ecotypes phenotyped in [22] are the parentals of the MAGIC [96]. This population consists of a large genetically-unstructured population of Recombinant Inbred lines (RILs) generated by inter-crossing 19 Arabidopsis parental lines and several generations of single seed descent [96]. The Arabidopsis MAGIC population displays a high degree of phenotypic variation in terms of rosette shape and size, making it suitable to dissect the genetics of complex phenotypic traits.

Here, we report machine-assisted acquisition of time-stamped images from MAGIC RILs during their rosette development and we extract a range of morphology descriptors for rosette size and shape variation. We continue the work of [22] and extend their approach to quantify and explore the underlying genetic basis of size and shape variation by a QTL mapping approach. Combined phenomics and genomics analyses identify 116 loci linked to the shape in the early developmental stages of Arabidopsis rosette growth.

## Material and methods

### Plant material

Phenotyping was performed on a core set of 485 RILs (3 replicates of each) from the MAGIC population [96, 97]. Seeds were obtained from the Nottingham Arabidopsis Stock Centre (NASC). Plants were previously genotyped by [96], using 1260 single nucleotide polymorphisms (SNPs) at the Illumina GoldenGate assay.

### Growth conditions

Experiments were performed in greenhouse chambers at the National Plant Phenomics Centre (NPPC) at IBERS, Aberystwyth University, UK. Seedlings were vernalized for 28 days at 5°C and 8h light/16h darkness cycle. This ensured that all genotypes germinated and flowered within the time course of the experiment.

Single seedlings were pricked out into 6 cm diameter pots (half filled with vermiculite and the upper half with 30% grit sand/70% Levington F1 peat based compost) and were transferred after 7 days to PlantScreen Phenotyping System (Photon Systems Instrument, PSI, Brno, Czech Republic) and grown under controlled conditions (18°C, 14/10h photoperiod, white light ~ 400 μmol m^-2^ s^-1^). Plants were imaged, weighed and watered to a predefined target weight of 65% of field capacity, daily, until most plants had flowered.

### Image acquisition, processing and morphology descriptors

Top view images were processed using an internal automatic workflow provided by the manufacturer. It performed the tasks of image processing, which calculated size and shape descriptors and stored them on the platform database. A detailed description of all descriptors can be found in [61] and Table 1. Rosette size was described by Projected Rosette Area (PRA) and Perimeter Length (PL). Rosette ground coverage comprised Compactness and Rotational Mass Symmetry (RMS) descriptors. Rosette deviation from a circle was measured with Slenderness of Leaves (SOL), Roundness (RND), Convex Hull Roundness (RCH), Isotropy (ISO) and Eccentricity (ECC).

**Table 1:** Morphology descriptors used in this study. Modified from PlantScreen User Manual, v1.5, 2017.

Group	Descriptor	Acronym	Description	Equation
Rosette size	Projected Rosette Area	PRA	Total area of visible plant surface. pixel count transformed into mm^2^	-
	Perimeter Length	PL	Boundary pixel count transformed into mm	-
Rosette coverage	Compactness	Compactness	Ratio between area and convex hull area. Convex Hull represents the smallest polygon surrounding the shape without any concavity.	$Comp.=\frac{Area}{{Area}_{Convex\;Hull}}$
	Rotational Mass Symmetry	RMS	Ratio between convex hull area outside and inside a circle with the same area (weighed by compactness) that the plant, which has its centre in the plant centroid	$RMS=\left(\frac{{Area}_{Circle\;Only}+{Area}_{Convex\;Hull\;Only}}{{Area}_{Intersection}}\right)$
Rosette geometry	Slenderness of Leaves	SOL	Ratio between the plant skeleton and area. The plant skeleton is a set of lines that runs through the medial axis, i.e. the central region, of plant leaves and petioles	$SOL=\frac{{Perimeter}_{Skeleton}^{2}}{Area}$
	Roundness	RND	Deviation from a circle using the relation between the area and the perimeter	$RND=4\cdot \pi \cdot \frac{Area}{Perimeter^{2}}$
	Convex Hull Roundness	RCH	Roundness calculated from the convex hull	$RCH=4\cdot \pi \cdot \frac{{Area}_{Convex\;Hull}}{{Perimeter}_{Convex\;Hull}^{2}}$
	Isotropy	ISO	Ratio of area and perimeter from a polygon, including leaf tips	$ISO=4\cdot \pi \cdot \frac{{Area}_{polygon}}{{Perimeter}_{polygon}^{2}}$
	Eccentricity	ECC	Shape elongation towards an axis. The spatial variance of plant pixels position is calculated. Then the mayor and minor axis of the ellipse with the same variation are computed.	$ECC=2*\frac{\sqrt[{2}]{{\left(\frac{1}{2}\cdot MajorAxisLength\right)}^{2}-{\left(\frac{1}{2}\cdot MinorAxisLength\right)}^{2}}}{Major\;Axis\;Length}$

### Phenotypic data analysis

All summaries and plots were performed using the R statistical computing environment [98]. Replicate values by Days After Sowing (DAS) were averaged and all calculations, including QTL mapping are performed using the mean value as representative of the RIL at given DAS.

Principal components analysis (PCA, function *prcomp* from the package *stats* [98]) was calculated to generate an uncorrelated shape space, i.e. to eliminate remaining size-effects and correlations among descriptors. PCA was built with the correlation matrix and all 9 Principal Components were retained so that RILs distances remain constant, given overall mean and variance scaling. Therefore, this PCA does not reduce dimensionality but constructs an uncorrelated morphospace with common aspects of rosettes grouped in each principal component. PCA was performed on RIL- averaged values rather than individual values.

**Fig 1:**
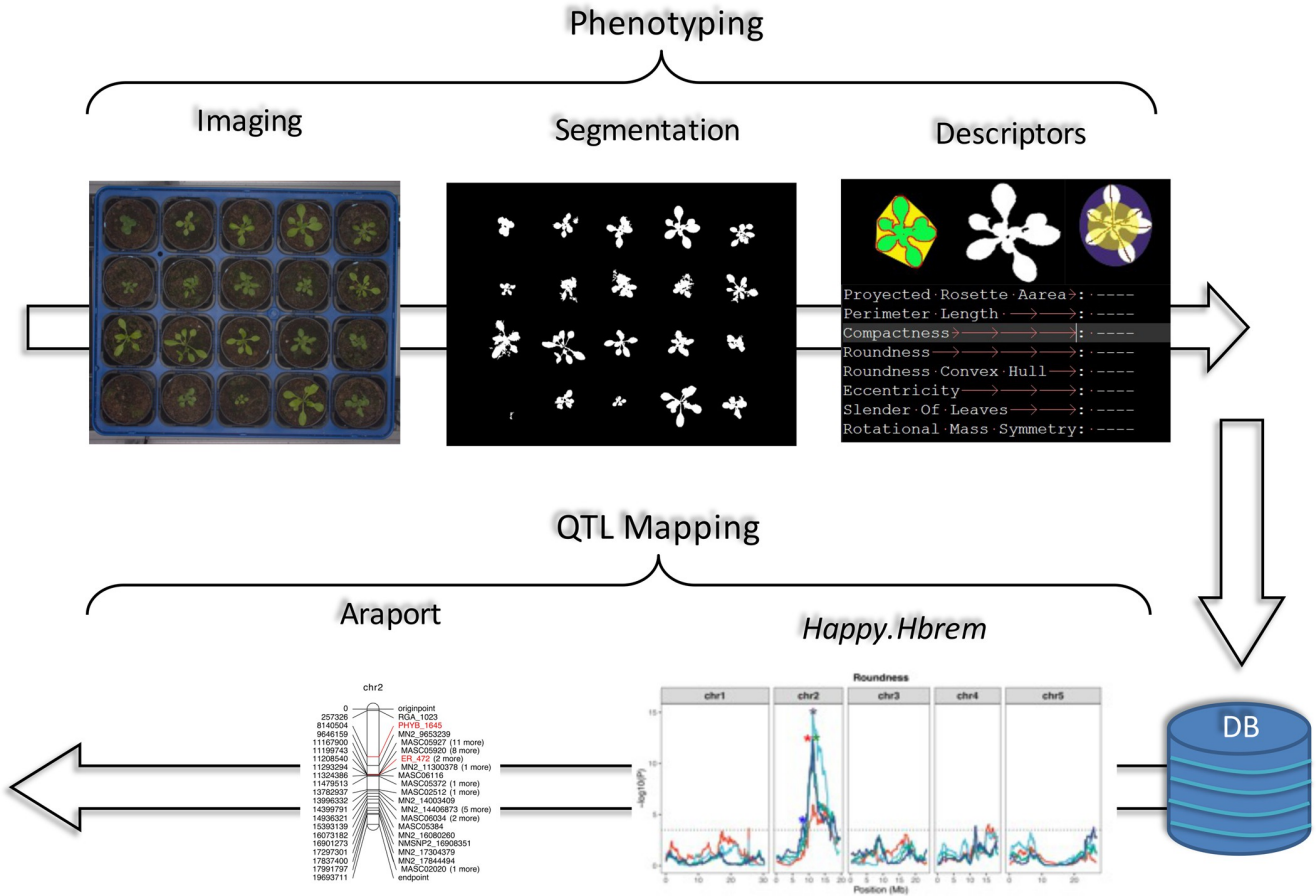
Workflow used in this study for phenotyping and QTL mapping. 485 RILs (3 replicates) were grown in 5x4 trays and imaged daily. Images were automatically segmented, rosettes were extracted, and analysed by device built-in pipelines. Shape descriptors and other metadata are recorded into a database. Shape descriptors and their PCA-derived morphospace were used for QTL mapping with *happy*.*hbrem* followed by gene search at ARAPORT11.

Pairwise Pearson’s correlation across descriptors’ averages were calculated at each DAS (function *cor* and *cor*.*test* from the package stats [98]). Broad-sense heritability (*H*^*2*^) was calculated independently at each DAS for all shape descriptors to estimate the proportion of the phenotypic variance explained by genetic variation. Variance decomposition random-effect models were fitted (function *lme* from the package *nlme* [99]) with phenotype (P_ij_) as dependent variable, RIL (R_i_ with I as line number) as a random factor and random residuals (ε_ij_) for each replicate plant j as *P_ij_* = *μ*+*R_i_*+*ϵ_ij_* [100]. Variance component estimates were extracted from the model with the function *varcomp* from the *ape* package [101]. Broad-sense heritability (*H*^*2*^) for each descriptor was estimated as $H^{2}=\frac{V_{g}}{V_{g}+V_{e}}$, where *V*_*g*_ is the variance among the RILs and *V*_*e*_ is the environmental variance. The general workflow for the data analysis is summarized in Fig 1.

### QTL mapping and candidate genes

Each shape descriptor (9 variables) and principal component (9 PCs) were split by DAS and used as input for QTL mapping with the *happy*.*hbrem* R package. This software was specifically designed for multi-parental population analysis [89] and previously used for Arabidopsis MAGIC population [96]. RILs genomes are reconstructed as parental haplotype mosaic (*happy*) working out the Identity By Descent (IBD) using a Hidden Markov Model [89]. For each phenotype, a genomic scan fits a Hierarchical Bayes random effects (*hbrem*) model [92] with 19 random factors, corresponding to founder alleles, weighed by the IBD probabilities. A permutation test randomizing phenotypes 500 times, established a genome-wide threshold for statistically significant QTL and corrects for multiple testing according to [96, 102]. Finally, a QTL location is defined as the peak marker with largest logP-value (from *hbrem* procedure) within an interval where other SNPs pass the genome-wide P-value (from the resampling procedure). A boxplot with allelic phenotypic effect for each founder ecotype was calculated using the *hbrem* procedure.

The amount of phenotypic variation explained by each marker was estimated using the package MagicHelpR (https://github.com/tavareshugo/MagicHelpR). The closest gene to the QTL marker was identified and gene annotations were retrieved from ARAPORT11 [103] using custom scripts.

The total number of traits was 180 comprising 9 shape descriptors and 9 PCs multiplied by 10 days. Some QTLs were found at several days and traits. To make the analysis tractable, a procedure to select non redundant QTLs was developed as follows. An R script goes through all QTLs, in order of date, from 35 to 44 DAS, and then by shape descriptor, following the order in Table 1 and PC1, PC2 … PC9. At each day and descriptor, the R script saved any QTL that was not reported before and removes redundant ones. Therefore, the significant QTL list was sorted, first by day and then by descriptor. Then, the relevance of each shape descriptor and the length of the phenotyping experiment can be evaluated by the number of QTLs found each day and per variable.

## Results

### Shape descriptors variation, heritability and correlation

**Fig 2:**
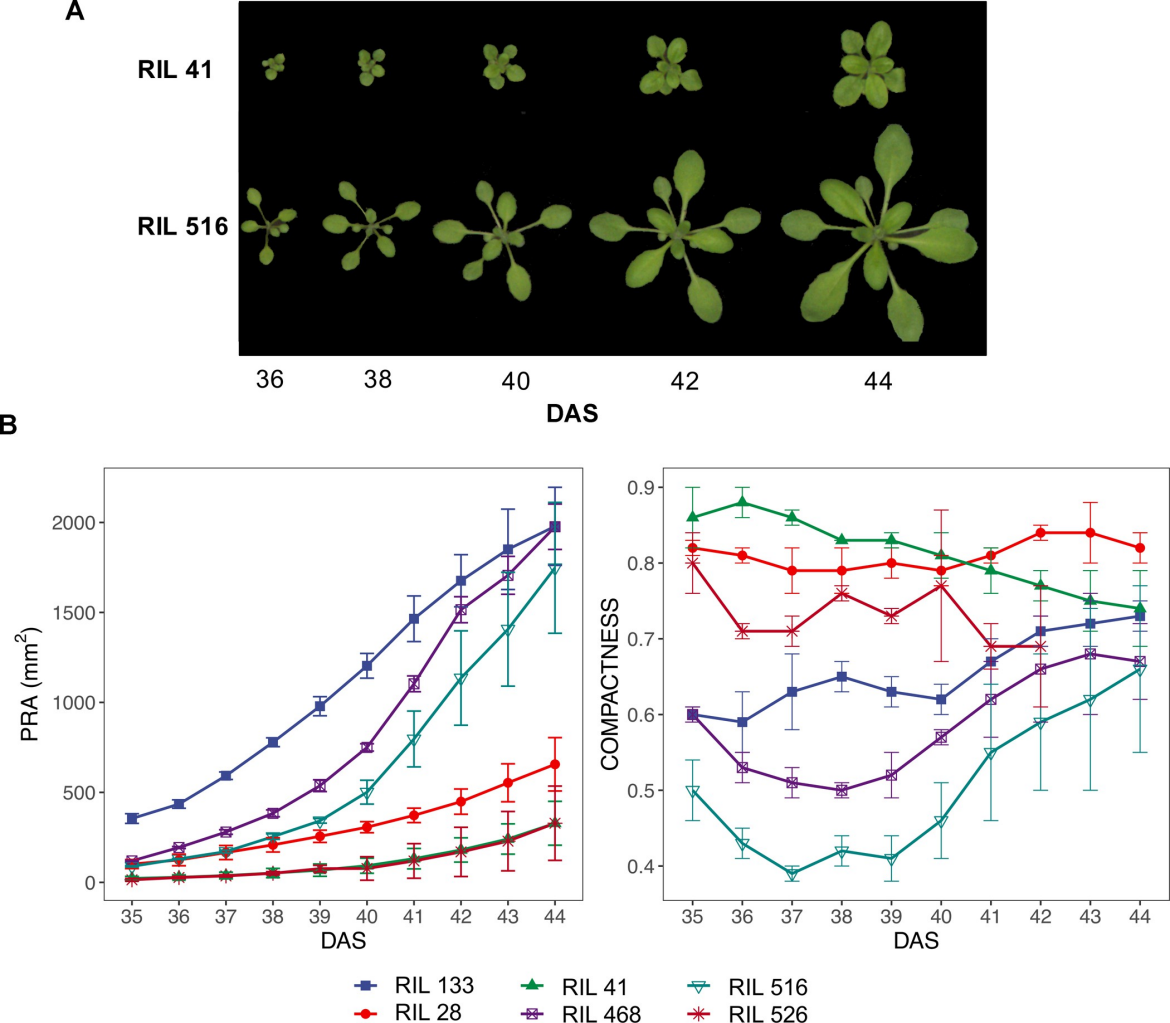
Rosette morphology variation of the MAGIC RIL population across time. **A.** Top view images of two contrasting rosettes, RIL 41 and RIL 516. **B.** Projected rosette area (PRA) and compactness from a set of six different RILs. DAS: Days after sowing. Error bars represent sample-based standard deviation (SD, n = 3). The six MAGIC lines have been chosen so they cover most of the range of phenotypic variation.

Cross comparisons across the RILs showed large variation in rosette morphology for the MAGIC RIL population. Fig 2 illustrates an example of six RILs and their values of Projected Rosette Area (PRA) and Compactness. S1 Fig includes the whole population and all traits and S1 Data contains averaged values per RIL for all nine shape descriptors and DAS. Rosette shape varied from genotypes with high surface coverage, short petioles and rounded leaves that do not extend far from the stem (e.g. RIL 41, Fig 2A) to genotypes with conspicuous gaps caused by longer petioles and / or elongated leaves that extend far enough to give a dispersed appearance (e.g. RIL 516, Fig 2A).

PRA showed values of 102 ± 57 mm^2^ (mean ± standard deviation) increasing daily between 25–35% up to an average of 1180 ± 400 mm^2^. RIL PRA variation had a min-max range of 385 mm^2^ at 35 DAS and 2239 mm^2^ at 44 DAS. Perimeter Length (PL) varied between 15 and 204 mm (mean ± sd: 78 ± 31mm) at 35 DAS increasing to a min-max range between 57 and 694 mm (mean ± sd: 353 ± 95 mm). The PRA-PL correlation (S1 Table) was 95% at 35 DAS, decaying through time down to 66.23%.

Compactness values were between 0 and 1 where 1 is a circle with no gaps and values lower than 0.3 represent a small circumference with no inner pixels, out of the range feasible for rosettes. At 35 DAS, compactness fluctuated between 0.38 and 0.86 (mean ± sd: 0.65 ± 0.07) with a similar range afterwards (mean values between 0.62 and 0.66). Compactness correlated negatively with PRA (-48.34% at 35 DAS) changing towards a slight positive correlation (3% at 41 DAS and 27.02% at 44 DAS). Compactness correlated negatively with PL (-65% at 35 DAS moving towards -36% at 44 DAS) (S1 Table).

ECC values vary from 0 (a circle) to 1 (a line). At 35 DAS, the rosettes had a range of values between 0.14 and 0.77 (mean ± sd: 0.34 ±0.09) decaying to a min-max between 0.08 and 0.30 (0.16 ± 0.03). ECC correlated negatively with PRA (around 50% on most DAS) and weakly with PL (between -16 and -54%). The correlation between ECC and compactness fluctuated between -32% and +15%. Eccentricity also correlated strongly with Rotational Mass Symmetry (RMS), over 80% between 35 and 38 DAS diminishing towards 60% at 44 DAS and 6% at 42 DAS. Correlation between ECC and roundness convex hull (RCH) was below -80% at all days except -74% at 40 DAS (S1 Table).

RMS (called rotational inertia and moment of inertia in rigid body physics) is also valued between 0 and 1. A value of 1 means an irregular rosette, either because it is eccentric or it is non-homogeneous. Small values of RMS indicate circular and high-coverage rosettes. RMS had values between 0.32 and 0.93 at 35 DAS (mean ± sd: 0.75 ± 0.1). RMS fluctuated with ample variation along days (min-max range around 0.70) between 0.12 and 0.77 at 44 DAS (mean ± sd: 0.42±0.10) (S1 Table).

RCH had low variation across time, with minimum values between 0.70–0.80 at all DAS and maximum values of between 0.92–0.97 with a dynamic range between 0.10 and 0.20. The correlation between RCH and RMS peaked at -66% at 35 DAS and increased towards -22% at 40 DAS and decreased again to -40% at 44 DAS (S1 Table).

RND values are between 0, circular rosettes, and 1 asymmetric ones. Rosettes RND ranged between 0.09–0.59 at 35 DAS, decreasing to 0.04–0.38 (mean ± sd: 0.22 ± 0.07 at 35 DAS to 0.13 ± 0.05 at 44 DAS). RND was negatively correlated with PL (below -70%) and with Slender of leaves (SOL) (-77.5% at 35 DAE) decreasing through time (-47% at 44 DAS) (S1 Table).

SOL had an unbounded dynamic range from 0 to 289 (in our rosette set). SOL minimum rose from 1.93 at 35 DAS to 4.24 at 44 DAS, and maximum also increased from 21 to 243. SOL had a strong positive correlation with PRA, around 78% at 35 and 36 DAS decreasing to 41% at 44 DAS (S1 Table).

Isotropy (ISO) was weakly but positively correlated with ***RND*** (50% to 61% from 38 DAS onwards) and negatively with PL (-51% to -67% between 38 DAS and 44 DAS) (S1 Table).

Heritability values (Table 2, S2 Fig) decreased across time as rosettes grew. Heritability for PRA, PL, Compactness and RND were over 50% at all times (RND baseline heritability was 48%). Heritability for SOL went from 62% at 35 DAS, at two cotyledons stage, to 49% at 38 DAS, with five or six leaves, and to 12% at 44 DAS. RCH, ECC and RMS had heritability values around 40% and went down to around 30% and to 10% for RMS. ISO had constant low heritability at all times, around 15–20%.

**Table 2:** Broad-sense heritability per morphology descriptor across time, showing the percentage of change between the last and first DAS.

Descriptor \ DAS	35	36	37	38	39	40	41	42	43	44
PRA	62	68	68	68	69	70	68	66	65	62
PL	67	74	75	72	72	69	62	58	46	47
Compactness	69	70	64	62	57	52	49	49	52	56
RMS	39	46	43	32	21	17	10	11	17	18
SOL	62	62	58	49	34	31	21	18	14	12
RND	64	70	67	61	59	55	48	46	43	48
RCH	47	44	43	41	35	34	27	29	28	29
ISO	16	23	22	23	26	25	23	22	17	21
ECC	39	48	52	47	37	37	18	27	28	28

Principal component analysis (S2 Table, S3 Fig) generated an uncorrelated morphospace for variation in rosette morphology. The first two principal components explained 75% of shape variation (51% PC1 and 24% PC2) in the RIL population. PC1 was a combination mainly of size components related to rosette age (according to the colour gradient from green to blue along time) and RND and ECC components. The second PC represents just shape components indicating that compactness is independent of size and correlating with RCH and RND, while these two did not correlate among them. These 2 PCs indicated that younger rosettes were quite eccentric and when they grew older they either got rounder or elongated in one direction. PC 1 is composed of PRA, PL, RCH and SOL as positive loadings and RMS and ECC as negative ones. PC2 is positively influenced by Compactness, RND, ISO and RCH, capturing the circularity of the rosette. PC3 (12% variance) is negatively influenced by Compactness, ECC and RMS and SOL, which are related with the asymmetry and the presence of inter-leaf gaps. PC4 (5% of variance) is dominated (0.84) by ISO. PC5 (4% of variance), positively weighted by SOL, RCH and RMS and negatively by PRA, accounts for small leaves spread out in a circular fashion. PC6 (2% of variance) is dominated positively by RMS and less by PRA and negatively by SOL, phenotyping large rosettes with gaps between leaves but leaf blade overlapping. PC7 (1% of variance) oppose Compactness and RND accounting for dense filled-in rosettes. PC8 and PC9 account for less that 1% of variance but PC9 identifies large rosettes with small perimeter, a sign of round shape.

### Dynamic QTL mapping

**Fig 3:**
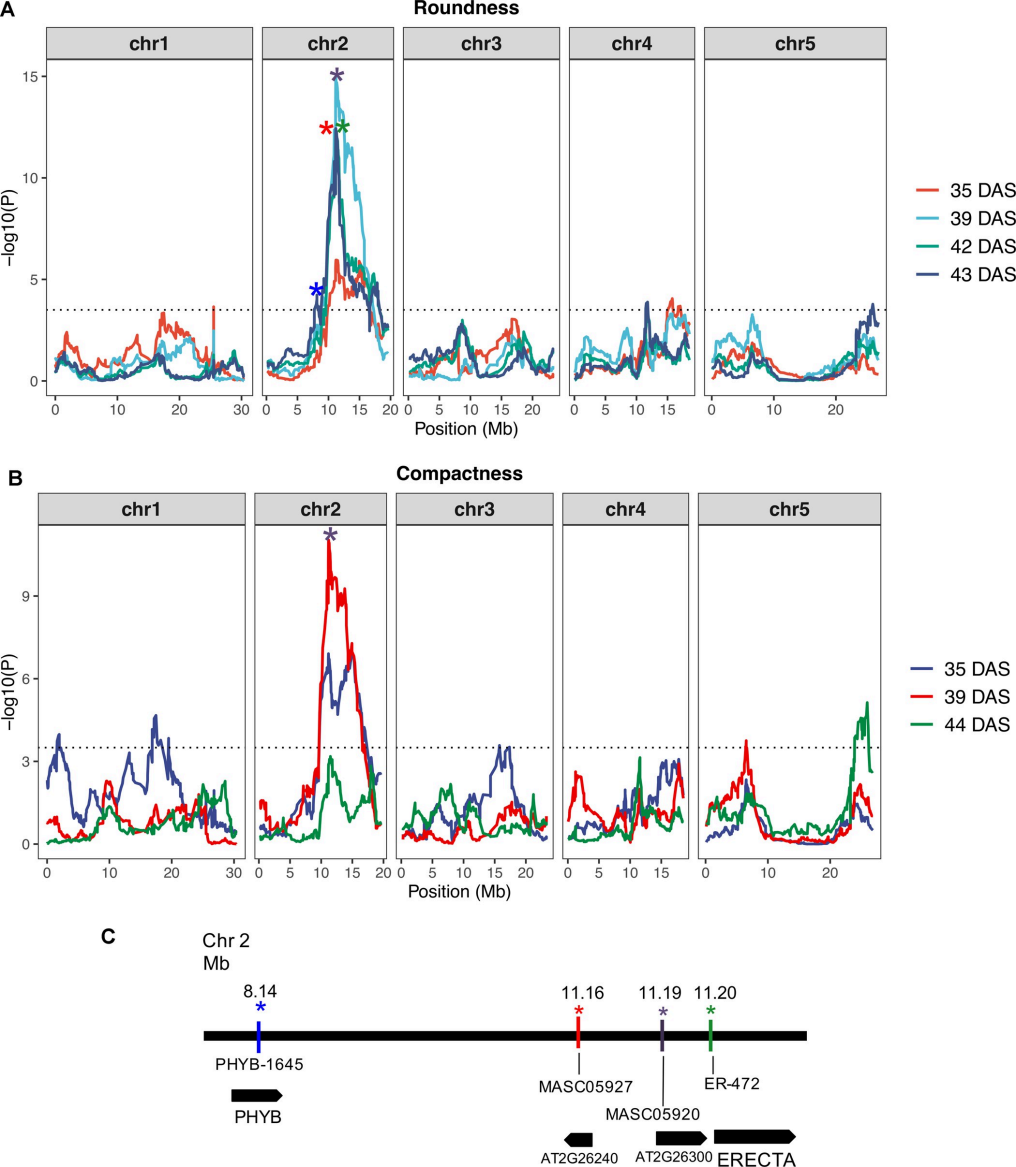
LOD score plots at different DAS. A. Roundness. B. Compactness. C. Close-up of chromosome 2 region surrounding PHYB_1645 SNP (blue asterisk) and ER_472 SNP (green asterisk). Gene models are shown under marker names. Significance threshold–log(P) ≥ 3.5 is shown as dotted lines.

A Bayesian multipoint QTL mapping [89, 91] was applied to all combinations of shape descriptors, including PCs, and DAS to find genetic markers associated with rosette morphological variation. This strategy identified 116 QTLs significantly associated (-log(P) ≥ 3.5) with phenotypic variation across time (S3 and S4 Tables, Fig 3). The physical position of markers queried in the ARAPORT 11 database for the closest gene accession, resulting in 105 candidate genes for shape variation in Arabidopsis rosettes (S5 Table).

Redundant associations (i.e. same locus found to be significant at several DAS or at several shape descriptors) were filtered out. Most QTLs were found either in the first 4 or in the last 3 DAS (Table 3). For shape descriptors (Table 4), Compactness, RND, PL, PC3 and PC2 contributed most to QTLs. Although PRA varied among rosettes, no associated markers were found even before filtering.

**Table 3:** QTLs found per day after removing redundant QTLs.

DAS	35	36	37	38	39	40	41	42	43	44
**# QTLs**	17	11	12	16	7	7	4	15	14	15
**Cum sum**	17	28	40	56	63	70	74	89	103	118

**Table 4:** QTLs found per variable after removing redundant QTLs. Organized by shape descriptor, sorted by QTL count.

Shape Descriptors	Compactness	RND	PL	PC3	PC2	RCH	ISO	ECC
**# QTLs**	29	23	10	9	8	6	6	5
**Cumulative sum**	29	52	62	71	79	85	91	96
**Shape Descriptors**	**PC4**	**PC6**	**PC8**	**PC1**	**PC5**	**PC9**	**PRA**	**SOL**
**# QTLs**	5	4	3	2	2	2	2	2
**Cumulative sum**	101	105	108	110	112	114	116	118

QTL tended to cluster on chromosome 2 at all DAS and mostly for compactness, RND and PL descriptors (Table 5, S3 and S4 Tables, S4 Fig). QTL at chromosome 1 were found mostly at 35 and 38 DAS and markers at chromosome 3, 4 and 5 were found mostly at 42 to 44 DAS. Estimated phenotypic values of the 19 parental alleles on compactness and RND are shown for the QTLs cluster on chromosome 2 (S5 Fig).

**Table 5:** QTLs found per chromosome after removing redundant QTLs.

Chromosome	1	2	3	4	5
**#QTLs**	23	54	13	13	15
**Cumulative Sum**	23	77	90	103	118

### Genes and markers associated to shape variation

S5 Table contains ARAPORT 11 gene accessions closest to QTL associated markers and are possibly related with the phenotype. The descriptions of these genes suggest that many regulatory genes related with hormonal and environmental signals may be related with the shape descriptors studied.

QTL on Chromosome 2 (Fig 3 and S4 Fig) were distributed across the whole chromosome with a dense cluster around the markers ER_472 and PHYB_1645. The highest p-values were found at markers MASC05920 and MASC05927 (p-values around 12 according to trait and day).

The marker MASC05920 was found in this region with maximum significance level (-log(P) between 10 and 15 according to date and descriptor) found for Compactness, RND and PC2 (S3 and S4 Tables). MASC05920 is located within AT2G26300 loci (gene name GPA-1). This gene encodes for a heterotrimeric G-protein alpha subunit involved in signal transduction (S5 Table). A BLASTP alignment between Arabidopsis and Saccharomyces GPA-1 (NCBI NP_011868) resulted in a 60% identity (positives), 55% identity (positives) with Caenorhabditis one (NCBI accession NP_001123018), indicating homology may be due to conserved motifs shared with most G-proteins. Comparisons of the distribution of haplotype effects at this QTL for both, compactness and RND, suggests that parental lines Ler and Can contribute mainly to the most compact and round rosettes; while the other parents have a similar distribution of effects (S5 Fig).

Other QTLs were associated with marker MASC05927, located in locus AT2G26240. This gene encodes for a transmembrane protein 14C (S5 Table) whose function has not been described so far. For this QTL, the Ler parental line is the main contributor to the RND phenotype. Marker ER_472 is located ~8.800 bp away from markers MASC05920 and MASC05927. This marker was significantly associated with RND at 42 DAS, explaining 15% of phenotypic variation (-*log(P)* = 12.1) (Fig 3A–3C). It was also found significant for ISO at 39 and 41 DAS, explaining between 6% and 12% of the phenotypic variation. ER_472 is a SNP within ERECTA gene (AT2G26330), which is annotated as a Leucine-rich receptor-like protein kinase family protein [104] and known to affect rosette shape [105–107]. The effect of the 19 haplotypes shows that the alleles conferring the largest RND effect are from Ler, followed by Can and Hi parental lines (S5 Fig). A QTL on chromosome 2 for RND was associated with marker PHYB_1645 (-*log(P)* = 4.2). This marker is located within the PHYB gene (AT2G18790) encoding for the PHYTOCHROME B gene (Fig 3A–3C), a photoreceptor sensitive to red:far red ratio. The effect of the 19 haplotypes for this QTL showed a similar, but no identical distribution of effects, with the Can parental line as the main contributor (S5 Fig).

Other relevant QTLs were found spread over the other chromosomes. A QTL on chromosome 5 (marker MN5_25963543) explained 8% of the phenotypic variation for RND and Compactness at 44 DAS (Fig 3A and 3B, S3 Table). Its closest gene is AT5G64930 (*CPR-5*), involved in plant defence (systemic acquired resistance—SAR). Four QTL were found in chromosome 4 associated with RND (S3 Table). Three of the genes identified encode un–characterized proteins, and the fourth encodes for the gene ATG4G22300 (*AtTIPSY1*).

## Discussion

Plant development occurs throughout the individual lifetime [108–110] and the production of new organs, i.e. stems, roots, leafs or flowers, is constantly influenced by the environment [111–113]. Ecotypes adapted to local environments often differ in many traits, particularly in the arrangement, size and shape of such organs. Research on phenotypic variation often refers to “morphology”, “form” and “shape” by implicit and informal definitions. As a consequence, the concept of shape can be reduced to adjectives like long-short, round-elongated, sparse-dense or into categories without explicit parameterization. Computer vision based shape descriptors are precisely defined, their measurement is objective, repeatable and interpretable as compared to visual human experience [22, 114, 115].

Extending the strategy previously used to describe shape variation of the 19 parental accessions in [22], we have quantified the rosette shape of 485 RILs. We used these measurements to associate genetic and phenotypic variation. Arabidopsis MAGIC population captures a reasonable range of natural variation and the inter-cross results in highly recombinant lines with higher mapping resolution to dissect quantitative traits genetic architecture [116] than biparental populations [117]. The lack of structure in MAGIC populations reduces false positives rates, which is an important drawback in association mapping [96].

The MAGIC population has been previously characterised for flowering time, height and fitness [96, 118] with a specific advanced statistical method for QTL mapping [89, 91]. This method takes advantage of multiple parents to assign genetic variants as multi-allelic markers rather than bi-allelic. In summary, MAGIC populations represent the best of both worlds, in the sense of high variation, low structure, high resolution and precision in the statistical methods. As a potential disadvantage the set of 485 RILs, with three replicates per RIL, became 1455 plants to keep under strict environmental control and daily phenotyping. To overcome this difficulty, a mechanised phenomics approach was necessary.

Our results support the idea that variation in rosette growth and shape involves multiple genes in a hierarchical control. This genetic structure should be able to exploit variable environmental conditions [119, 120], regulate heterochrony [109, 121], and enable a phenotypic response to variation in vernalization and photoperiod, e.g., flowering time or branching pattern.

Shape descriptors were correlated, suggesting ecologically related trait syndromes [122]. Therefore, they can be grouped into functional traits. PRA, PL, and SOL form a cluster capturing information on size and length. ECC and RMS form another cluster describing rosette elongation. RND and ISO describe the pattern of leaf arrangement as in a circle or a star-like shape. A singleton comprising only RCH captures accurately the closeness of rosettes to a perfect cycle, regardless of the gaps between leaves. Compactness would be another singleton describing rosette coverage.

Significantly associated markers were found using a dynamic QTL approach with a full combination of shape traits and DAS. This method yields up to 180 variables to test, thus increase the analytic effort with respect to single time point and single variable approaches, yet also increase the number of QTLs that would otherwise not be found using these other common strategies, e.g. phenotyping rosette size at the six leaves stage.

The markers found at chromosome 2 form a region with several potential genes related to rosette morphology. A first example is the GPA-1 gene. In yeast, the GPA gene is related to signal transduction in pheromone response pathway [123]. In Arabidopsis, amongst other functions, it is related to blue light induction of phenylalanine production [124], abscisic acid responses [125] and modulation of hypocotyl elongation and leaf formation (recessive mutants show round leaves and elongated petioles associated to sugar signalling and response-associated cell death [126]. GWAS association with environmental variables in the 1001 genomes population found SNPs markers related with the γ-subunit of a heterotrimeric G-protein, AGG3, related with cold tolerance [8]. The AGG3 protein is related to seed and organ growth [127] and shape [128], connecting this activity with the single G-protein alpha subunit found in Arabidopsis, GPA-1 and their orthologs in rice. GPA-1 also regulates germination, seedling development, reaction to environmental changes and stomata opening by means of ABA signalling. Mutants for GPA-1 are sensitive to ABA signalling [129]. ABA, together with ethylene and gibberellins, affect phenotypic plasticity related variation in leaf architecture [130]. This candidate alone would support a highly significant QTL in this region but there were other QTLs close to this marker that may be novel. For example, the gene AT2G26240 encoding for the transmembrane protein 14C is suspected to be related with fatty acid transport, FAX7 fatty acid export 7 [131]. The gene ERECTA (AT2G26330) is involved in shade avoidance responses (SAS) and the general morphology [132]. The gene PHYB is a well-known photoreceptor involved in the shade avoidance syndrome (reviewed in [133]), playing an important role in canopy development and morphology [134]. The gene CPR-5 (AT5G64930) is not directly related with morphology but with plant defences, yet, changes in rosette shape have been reported in *cpr5* mutants in response to light and altered salicylic acid levels [135, 136]. Another gene close to a marker QTL and involved in plant defence is ATG4G22300 (*AtTIPSY1*) [137].

Overall, from the 116 markers significantly associated with the rosette shape descriptors, most are located within loci with known or suspected regulatory functions (S5 Table). These were several membrane proteins and receptors, including a G-protein subunit like *GPA-1* (AT2G26300), a protein kinase receptor like ERECTA (AT2G26330), transcription factors like, *PHYTOCHROME RAPIDLY REGULATED1 (PAR1*, AT2G42870*)*, or chromo proteins like PHYB (AT2G18790) and hemoproteins, like HO2 (AT2G26550), participating on photomorphology and shade avoidance responses, by regulation of an auxin-responsive gene [138] and similar environment response phenotypes.

This study focuses on the global shape of an organ assemblage, the *A*. *thaliana* rosette. Rosette leaf shape varies along the ontogenetic development according to their local environment [67, 139], e.g., in a single plant some leaves are longer than others, usually towards light sources, with crenate, undulate or entire borders. Yet, rosette appearance is distinguishable among ecotypes and similar within individuals of the same ecotype, especially when grown in homogenous conditions [22]. Thus, rosette shape variation is visible and genetically controlled within some range as predicted by the ‘continuum and process morphology’ [140–142]. Our results on shape descriptors heritability are consistent with these observations and build up on the concept that morphological traits act as functional ones [63, 143]. The QTLs found here agree with the theory that finely tuned genetic regulatory networks, linking and integrating environmental clues during ontogenetic development, are among the major contributions to plant local adaptations [144–147]. In this sense, our study introduces the automated plant phenomics as a relevant tool for the so called eco-evo-devo [148] with particular emphasis on morphology at a subspecies taxa level [149, 150]. From an applied biology perspective, the QTLs reported may be useful for further research either in their role on phenotypic regulation or the type of genetic variants they bear. For example, it can be argued that genetic manipulation of phytochromes or kinase receptors may potentiate crop adaptability to extreme environments or reduce undesirable variation due to early stage disturbances like short-term frost, drought or salt stress [15].

## Supporting information

S1 Fig Rosette descriptors through time for the 485 MAGIC RIL population and their principal components. Each line represents a RIL. Bold lines remark 8 random RILS.
(TIFF)

S2 Fig Broad-sense heritability per morphological descriptor across time. (TIFF)

S3 Fig Principal components analysis biplot. PC1 vs PC2 coloured by DAS (35 DAS: Green, 44 DAS: Blue).
(TIFF)

S4 Fig Significant markers for all morphological descriptors across time at five Arabidopsis chromosomes. In red ERECTA and PHYB markers. Number in parenthesis besides each marker means the number of times the same marker was identified for all descriptors across time.
(TIFF)

S5 Fig Boxplots with the estimated values of 19 parent alleles on roundness and compactness for the main QTL detected. Four QTLs on chromosome 2 at 39, 42 and 43 DAS are shown for roundness, and one QTL for on chromosome 2 at 39 DAS is shown for compactness.
(TIFF)

S1 Table Correlation pairs between morphology descriptors across time in DAS. Grey background cells indicate values between -50% and 50% and red coloured values indicate negative correlations.
(XLSX)

S2 Table Principal component analysis loading values for the whole set of shape descriptors through time, scaled by mean and standard deviation and calculated over the correlation matrix. Grey background cells indicate values between -0.5 and 0.5 and red coloured values indicate negative loadings.
(XLSX)

S3 Table Set of QTLs identified in all the morphology descriptors across time. Including the region position of the QTL, the position of the SNP peak, the significance, *p-value*, percentage of variation.
(XLSX)

S4 Table Set of QTLs identified in all the PCA based descriptors across time. Including the region position of the QTL, the position of the SNP peak, the significance, *p-value*, percentage of variation.
(XLSX)

S5 Table Set of Gene accessions at ARAPORT 11 closest to the QTLs found. It contains the closest gene to every SNP peak, its gene annotation, position and the count of combinations DAS and descriptor for which the SNP peak was significantly associated to the marker each gene accession is the closest.
(XLSX)

S1 Data RIL-averaged rosette descriptors data for the 485 MAGIC RILs across time and principal components. Three replicates per RIL were phenotyped for the 9 shape descriptors at Table 1 and their RIL group means were recorded for further analysis. Principal Components extracted from the correlation matrix–all DAS and RILs pooled–where calculated from RILs averaged phenotypic values. Colour code shows the trajectory of traits along time where every variable has been code in green for low values, yellow for intermediate and red for large. The color code is calculated for all DAS and RILs and recalculated for every feature. Statistics per DAS are recorded at the bottom of the table.
(XLSX)
