# Supplementary figures and images for: Genetic architecture of variation in Arabidopsis thaliana rosettes
Source: PLoS One. 2022 Feb 16;17(2):e0263985. doi: 10.1371/journal.pone.0263985 (PMC8849614; doi:10.1371/journal.pone.0263985)

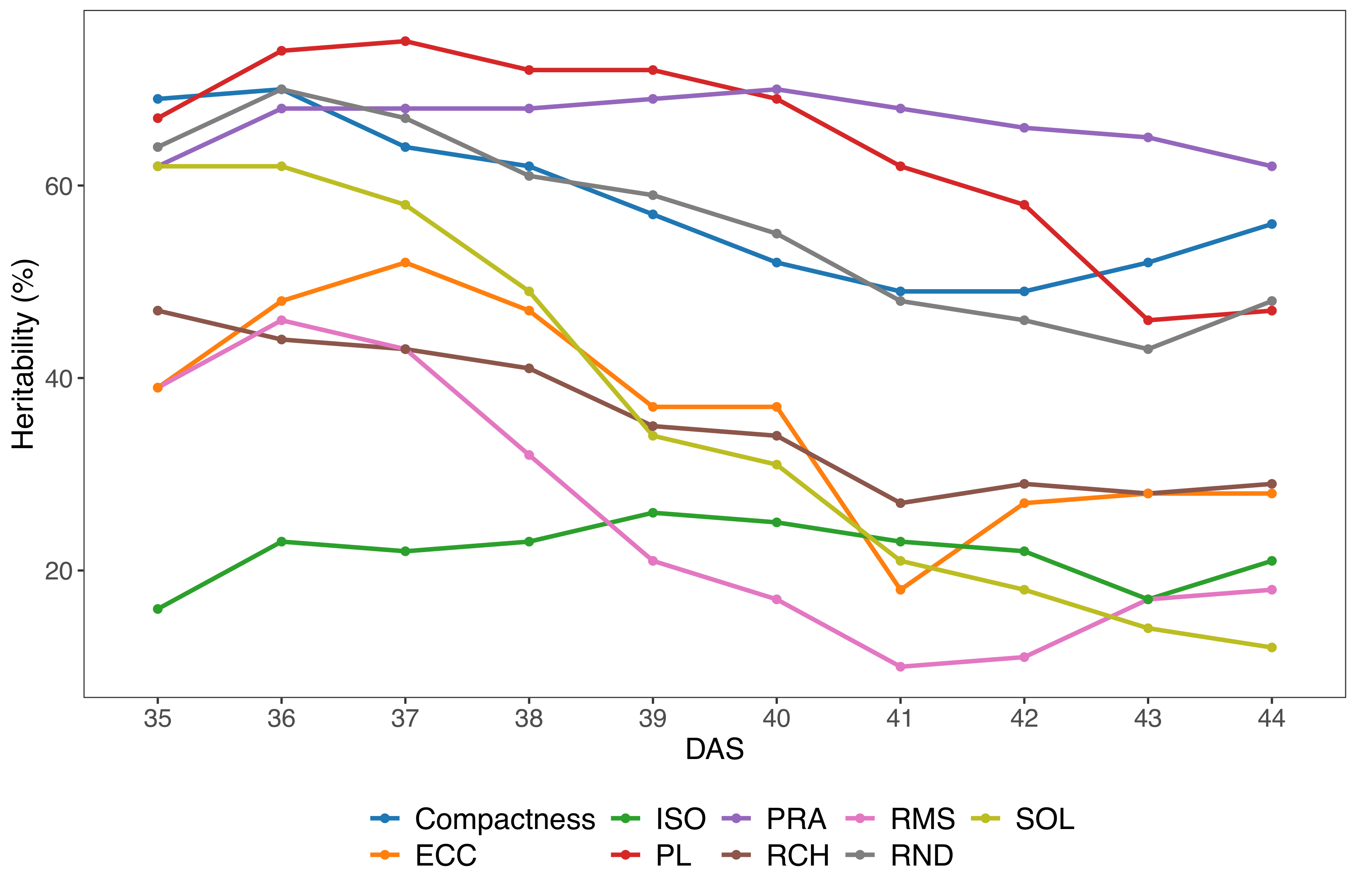

Supplement: S2 Fig — (TIFF) [file pone.0263985.s002.tiff]

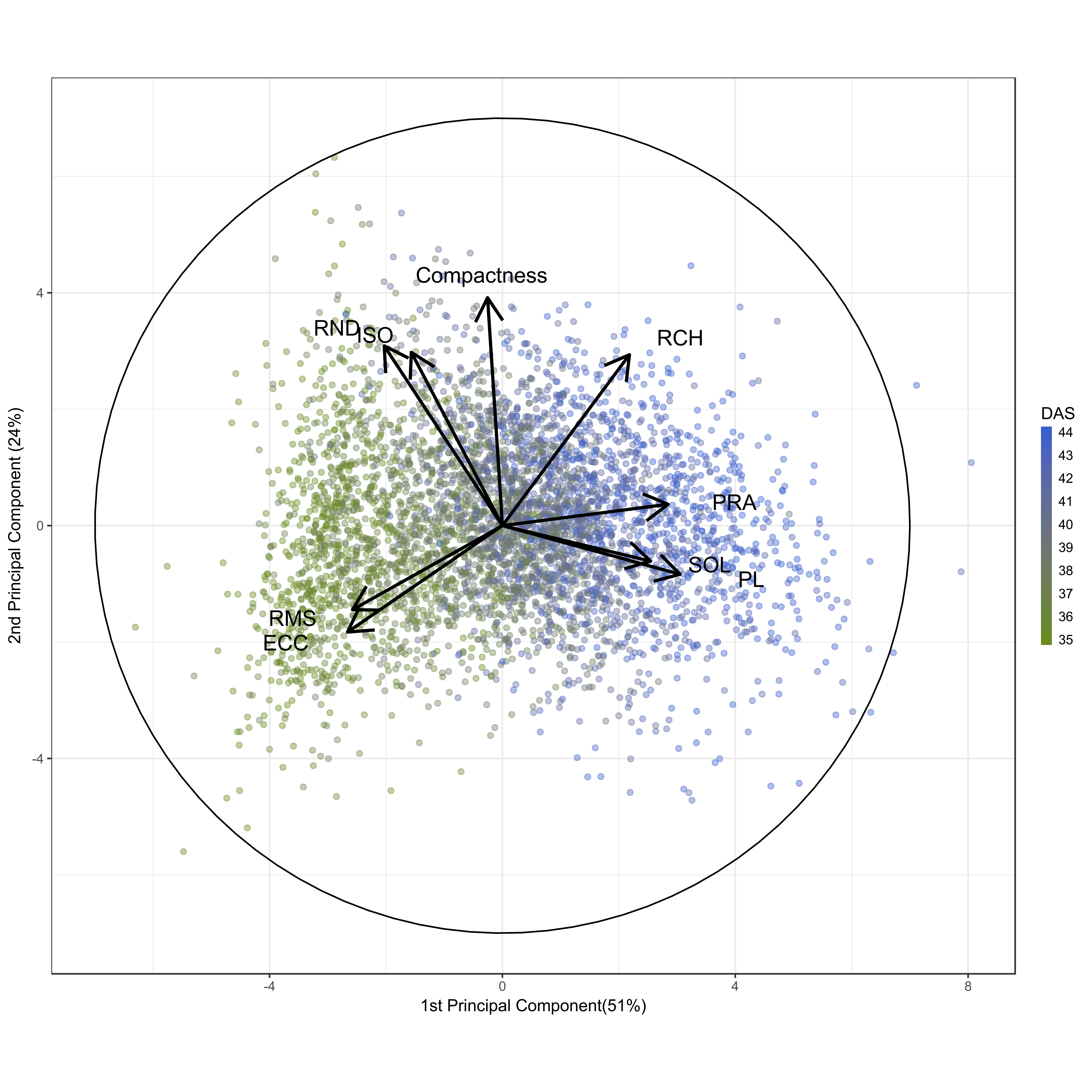

Supplement: S3 Fig — PC1 vs PC2 coloured by DAS (35 DAS: Green, 44 DAS: Blue). (TIFF) [file pone.0263985.s003.tiff]

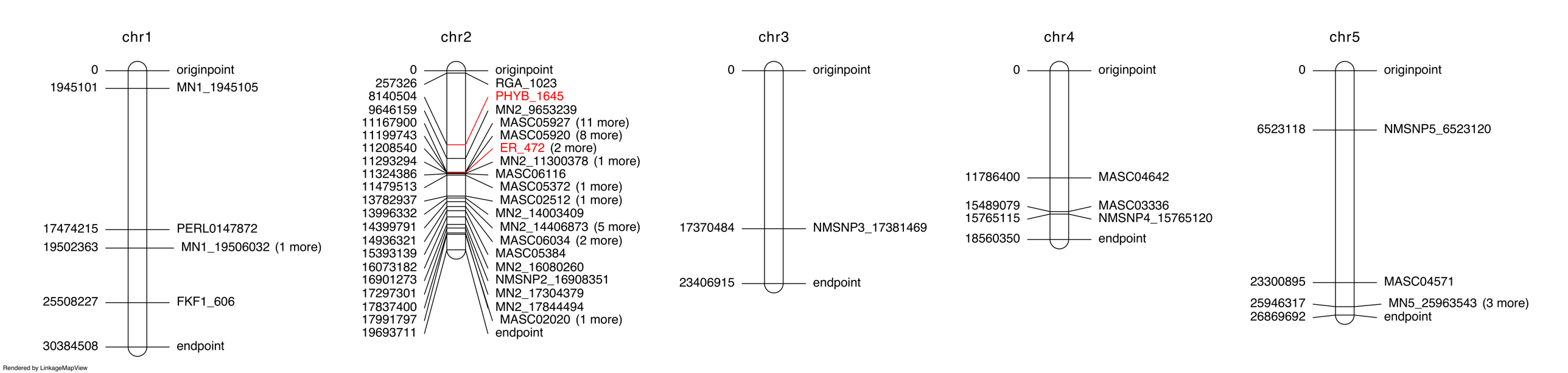

Supplement: S4 Fig — In red ERECTA and PHYB markers. Number in parenthesis besides each marker means the number of times the same marker was identified for all descriptors across time. (TIFF) [file pone.0263985.s004.tiff]

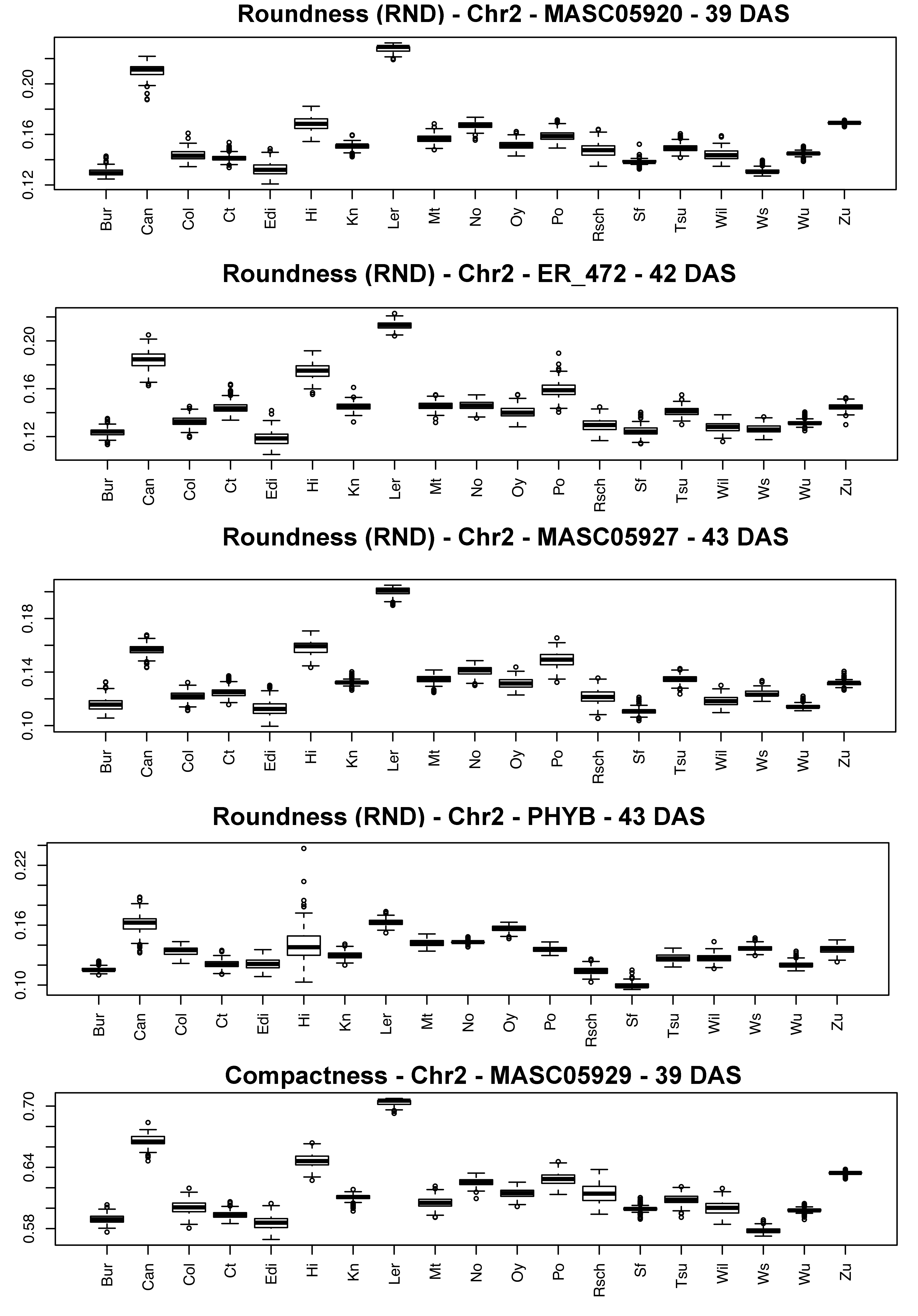

Supplement: S5 Fig — Four QTLs on chromosome 2 at 39, 42 and 43 DAS are shown for roundness, and one QTL for on chromosome 2 at 39 DAS is shown for compactness. (TIFF) [file pone.0263985.s005.tiff]
